# Crystallization of nickel sulfate and its purification process: towards efficient production of nickel-rich cathode materials for lithium-ion batteries

**DOI:** 10.1039/d3ra04280d

**Published:** 2023-09-27

**Authors:** Kyoung Hun Choi, Gisele Azimi

**Affiliations:** a Laboratory for Strategic Materials, Department of Chemical Engineering and Applied Chemistry, University of Toronto 200 College Street Toronto ON M5S 3E5 Canada; b Department of Materials Science and Engineering, University of Toronto 184 College Street Toronto Ontario M5S 3E4 Canada g.azimi@utoronto.ca

## Abstract

NiSO_4_·6H_2_O is an important salt for the battery-making industry. The extraction of nickel sulfate relies on the hydrometallurgical processing of nickel ores as well as the recycling of nickel-containing products. The last step in hydrometallurgical processing is the crystallization of nickel sulfate. Because of the similar ionic radius and ionic charge between nickel and magnesium ions, magnesium undergoes isomorphous substitution and replaces nickel ions in the crystal lattice structure of NiSO_4_·6H_2_O. This poses a challenge as achieving the desired metal salt purity is difficult, resulting in an inferior cathode material for nickel-containing batteries. In this work, the removal of magnesium during the purification process of NiSO_4_·6H_2_O crystals *via* a repulping process was thoroughly investigated. Moreover, the impurity uptake mechanisms of magnesium into NiSO_4_·6H_2_O crystals were investigated. The results indicated that repulping NiSO_4_·6H_2_O crystals with a saturated NiSO_4_ solution results in 77% removal of magnesium. Using a second-stage repulping process is less effective with only 26% magnesium removal. The purification efficiency of the two repulping stages was quantified by the equilibrium distribution coefficient, which corroborates the trend of decreased removal of magnesium in the second stage of repulping compared with the first stage. The primary impurity uptake mechanisms of magnesium into NiSO_4_·6H_2_O crystals were identified to be surface adsorption and lattice substitution (isomorphous substitution).

## Abstract 2

The distribution coefficient (*D*_2/1_) for magnesium in Ni_S_O_4_·6H_2_O crystals, a crucial salt used in battery manufacturing is assessed. The research also explores the efficiency of repulping and washing techniques in eliminating impurities from the crystals.

## Graphical abstract

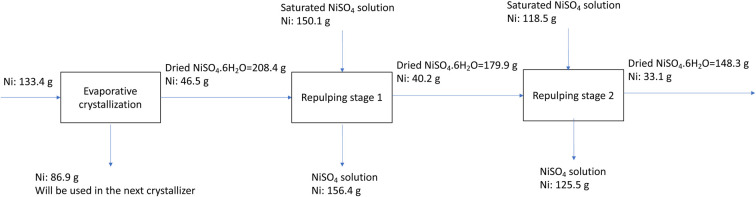

## 1. Introduction

Global challenges including climate change, diminishing natural resources, and urbanization necessitate a carbon-neutral energy source that can sustain global economies while protecting the environment. Consequently, there is a global push toward renewable energy and the electrification of transportation. This, in turn, increased the global demand for lithium-ion batteries, as the global lithium (Li)-ion battery market was valued at $29.86 billion in 2017 and is estimated to reach $139.36 billion in 2026 due to this increase in demand.^1^ To produce lithium-ion batteries with higher energy density, longer cycle life, and improved safety, cathode materials of Li-ion batteries have been the hotspot of much ongoing research. Out of the several cathode materials that have been developed in the last three decades, lithium nickel manganese cobalt oxide (Li-NMC) has shown promising results in terms of specific capacity, as it delivers a specific capacity of 180–200 mA h g^−1^ compared with 150 mA h g^−1^ obtained from LiCoO_2_.^2^ This advantage has led to its domination in the utilization of electrical vehicles (EVs), power tools, and medical and portable devices despite its relatively late commercialization in 2004.^3^

As the fifth most abundant element on the Earth's crust, nickel (Ni) is used in over 300 000 products^4^ including nickel-containing cathode materials such as Li-NMC. Battery-grade nickel used in the NMC cathode material is usually in the form of nickel sulfate hexahydrate (NiSO_4_·6H_2_O).^5^ To obtain high-purity nickel sulfate, hydrometallurgical processing of primary sources such as lateritic nickel ores and nickel sulfide minerals,^6^ or secondary sources such as spent nickel-containing lithium-ion batteries^7^ is often employed. The general hydrometallurgical processing involves pretreatment, acid leaching, separation/purification, and crystallization.^5^

The final step of hydrometallurgical processing, crystallization, is a process of forming solid crystals from a solution driven by the degree of supersaturation in the system. A supersaturated state refers to a condition where excess solutes are dissolved in the solution compared with the equilibrium condition. Hence, at constant temperature and pressure, the condition when crystallization occurs can be described by the chemical potential difference between the out-of-equilibrium state of the solute and the corresponding equilibrium state:^5^1
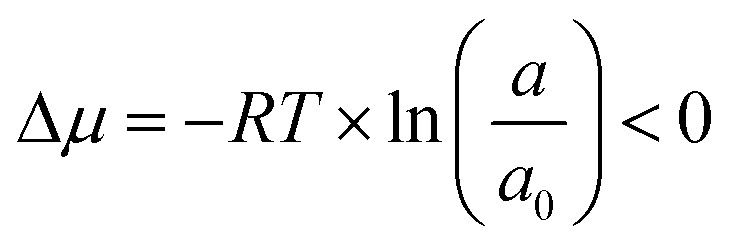
where Δ*μ* is the chemical potential difference of the solute, *R* is the universal gas constant, *T* is the absolute temperature (*K*), *a* is the activity of the solute in the solution, and *a*_0_ is the activity of the solute in the solid phase at equilibrium. As shown in eqn (2), the activity ratio can also be approximated by the ratio of concentrations, where *C* is the concentration of the solute and *C*_eq_ is the solubility of the solute. With this relationship, the chemical potential can be described as a function of both concentration *C* and solubility *C*_eq_ at constant temperature and pressure, and the driving force for crystallization can be presented by the supersaturation ratio *S*, where supersaturation occurs when *S* > 1:^5^2
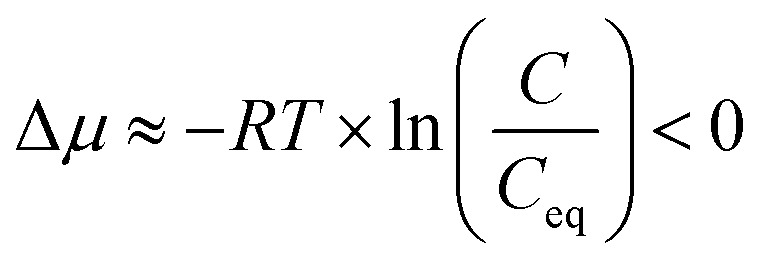
where3
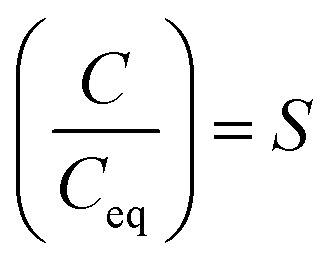


Many industrial crystallization techniques can achieve supersaturation. In evaporative crystallization, the supersaturation increases by removing the solvent through evaporation. The generated supersaturation in the system will be then consumed by nucleation and growth of crystals.^8^ While evaporative crystallization removes most of the impurities, previous studies have indicated that magnesium is a notable impurity captured during nickel sulfate evaporative crystallization.^9^ This poses a problem in the battery-making industry as magnesium negatively affects the Li-NMC cathode material, even when present at low concentrations. For instance, it has been reported that the magnesium content of *x* > 0.01 in the Li[(Ni_1/3_Co_1/3_Mn_1/3_O_2_)_1−*x*_Mg_*x*_]O_2_ cathode material was detrimental to the capacity of the cathode material.^10^ Another drawback of evaporative crystallization includes high working temperatures, requiring relatively large energy consumption.^11^ Thus, several alternative approaches for nickel sulfate crystallization have been proposed, which include eutectic freeze crystallization, cooling crystallization, and antisolvent crystallization.^5,12^ However, none of them have been applied in the industry due to their limited empirical data to ensure efficient production of NiSO_4_·6H_2_O and their requirement for additional solvents/reactions. Despite the drawbacks of the conventional evaporative crystallization method, it has the advantage of a relatively high crystal growth rate, well-established procedures, and no requirement for additional solvents/reactions.^5,11^ Therefore, this study focuses on developing a purification process of NiSO_4_·6H_2_O crystals that is in conjunction with evaporative crystallization.

In the hydrometallurgy industry, the repulping stage is often employed to remove impurities that are incorporated as a result of surface adsorption/absorption and the inclusion of an impurity-laden mother liquor.^13^ The repulping stage involves washing the crystals in their saturated solution to remove the impurities.^14^ However, no data have been reported on the purification efficiency (*i.e.*, the extent of impurity removal) of the repulping of NiSO_4_·6H_2_O crystals. It is imperative to acquire these data to provide valuable insights into refining the purification process of NiSO_4_·6H_2_O crystals.

As previously described, magnesium is a notable impurity that is captured during nickel sulfate crystallization. Previous studies^15,16^ suggest that the formation of mixed crystals can be contributed to the isomorphic substitution of magnesium ions (Mg^2+^) into the crystal lattice structure, owing to the similar ionic radius and charge between magnesium ions and nickel ions (Ni^2+^). However, no extensive fundamental study has been conducted to strategically investigate the possible mechanisms of magnesium incorporation. This lack of knowledge poses a challenge to the industry, as understanding the exact mechanism of impurity incorporation is fundamental in controlling and designing the crystallization system to obtain crystals with higher purity. Thus, this study focuses on the investigation of different mechanisms of magnesium incorporation into NiSO_4_·6H_2_O crystals.

In this study, evaporative crystallization of NiSO_4_·6H_2_O crystals from an industrial nickel sulfate solution was carried out, followed by the subsequent repulping steps as additional purification steps. The purification efficiency of each stage was assessed by evaluating the distribution of magnesium in the solid phase and the liquid phase during the repulping stage. Additionally, the emphasis was put on identifying the mechanisms of magnesium uptake into NiSO_4_·6H_2_O crystals *via* a systematic investigation of the possible methods of incorporation.

## 2. Experimental

### 2.1. Materials

The chemicals used in the experiment were NiSO_4_·6H_2_O (purity ≥ 98%, Thermo Scientific), MgSO_4_·7H_2_O (purity ≥ 98%, Thermo Scientific), NaSO_4_ (purity ≥ 99%, Thermo Scientific), CuSO_4_·5H_2_O (purity ≥ 98%, Thermo Scientific), ZnSO_4_·7H_2_O (purity ≥ 99%, Thermo Scientific), CaSO_4_ (purity ≥ 99%, Thermo Scientific), and deionized water produced using a Milli-Q Integral water purification system (0.055 μS cm^−1^, MiliporeSigma, Merck KGaA, Darmstadt, Germany).

### 2.2. Equilibrium distribution coefficient

As one of the principal impurity incorporation mechanisms, partial solid solutions or mixed crystals are formed because of isomorphic substitution.^17^ Isomorphic substitution replaces an ion in the crystal lattice structure with a foreign ion with similar chemical and structural properties. The distribution coefficient, *K*_i_, is the first quantitative parameter of isomorphous substitution, and it is in accordance with the Berthelot–Nerst distribution law:^18^4
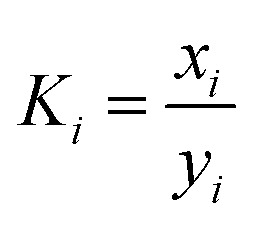
where *x*_*i*_ and *y*_*i*_ are the concentrations of component *i* in the solid and liquid phases, respectively. Following the conditions of the Berthelot–Nerst distribution law, the distribution coefficient is valid at thermodynamic equilibrium, where the true equilibrium between the solid and liquid phases must be achieved to remove all concentration gradients. Crystallization processes are usually realized under non-equilibrium conditions, and consequently result in inconsistent values of the distribution coefficient of impurities. As a response to this problem, Chlopin proved the method of achieving thermodynamic equilibrium between mixed crystals and solution, based on the recrystallization phenomenon.^18^ This enabled the determination of the equilibrium distribution coefficient, 
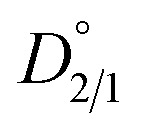
, given by eqn (5):5
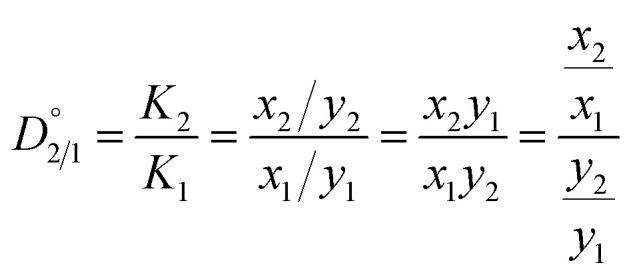
where *K*_1_ and *K*_2_ are the distribution coefficients of the host and the impurity ion, respectively. The lower value of 
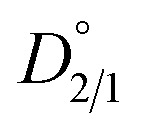
 signifies that the impurity ion partitions favorably into the liquid phase under the equilibrium condition, leading to the rejection of impurities from the crystals. In contrast, a higher value of 
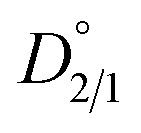
 indicates the impurity ion partitions favorably into the solid phase than in the liquid phase at equilibrium, thereby resulting in the uptake of impurity in the crystalline material.

### 2.3. Experimental procedure

#### 2.3.1. Synthetic solution preparation

Table 1 summarizes the composition of the synthetic nickel sulfate solution based on a real industrial solution provided by our collaborator. A 1.5 L synthetic solution of NiSO_4_·6H_2_O with known concentrations of impurities was prepared with deionized water. The solution was prepared in an Erlenmeyer flask immersed in a water bath (Fisher Scientific, Inc.). The solution was heated to 70 °C to ensure the complete dissolution of a reagent before adding the next reagent. After diluting the solution to 1.5 L with deionized water, the synthetic solution was cooled down to 50 °C. The concentration of the metal ions in the synthetic solution was measured by inductively coupled plasma optical emission spectroscopy (Optima 8000, PerkinElmer) at the following wavelengths: K 766.490 nm, Ca 317.933 nm, Cu 327.393 nm, Zn 206.200 nm, Mg 280.271 nm, Na 589.592 nm, and Ni 231.604 nm.

**Table 1:** Composition of the industrial nickel sulfate solution [g kg^−1^]

Sample	[g kg^−1^ solution]						
	Ca	K	Na	Cu	Mg	Ni	Zn
Intended solution	0.02	0.02	0.004	0.12	0.87	74.00	0.64
Actual solution	0.02	0.01	0.007	0.21	0.87	70.11	0.55

#### 2.3.2. Evaporative crystallization

**Fig. 1:**
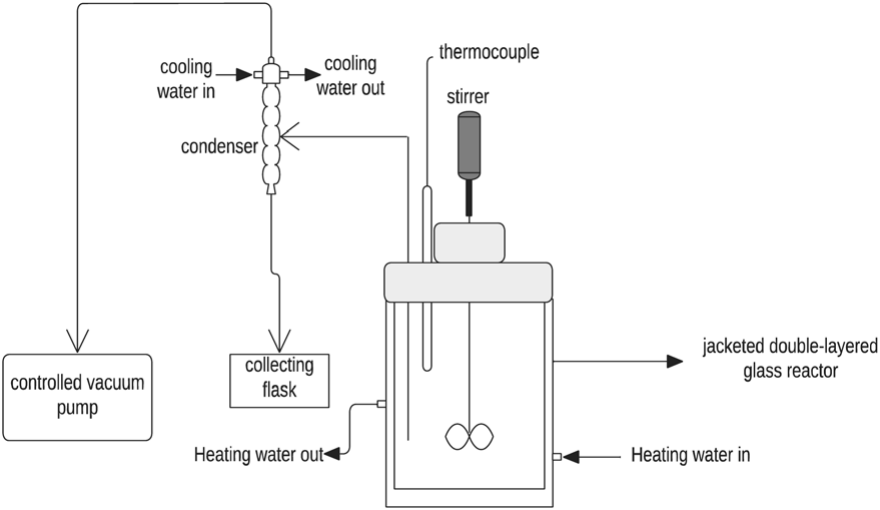
Schematic diagram of the experimental setup of semi-batch evaporative crystallization.

Semi-batch evaporative crystallization was carried out in a 2 L jacketed glass reactor with a Liebig condenser, a receiving flask, a mechanical stirrer, and a control panel that established the rotational speed of the stirrer to 150 rpm (JGR-2L, YH CHEM). A vacuum pump equipped with a manual regulator (VP18R, Laboratory Supply Network) was used to control the pressure inside the crystallizer. A Liebig condenser and a receiving flask were used for vapor condensation and receiving distilled water from the crystallizer. A water bath (Fisher Scientific, Inc.) was used to circulate hot water around the jacket layer of the reactor to maintain a temperature of 50 °C inside the reactor. Likewise, a chiller (Fisher Scientific, Inc.) was used to circulate the coolant around the condenser. A schematic diagram of the experimental setup can be shown in Fig. 1. According to the industrial practice, the vacuum evaporation step was carried out until 60% of the water from the initial synthetic solution evaporated. After the vacuum evaporation step, the vacuum was removed, and the slurry was left to stir overnight at atmospheric pressure and 50 °C. After stirring overnight, NiSO_4_·6H_2_O crystals were obtained by vacuum filtration and washed with deionized water (weight of the deionized water equivalent to 10 wt% of wet crystals). The vacuum filter apparatus was pre-heated to 50 °C to avoid undesired crystallization. After vacuum filtration, the wet solids were dried inside the vacuum desiccator for 48 h.

#### 2.3.3. Repulping tests

According to the industrial practice, about 100 g of dried NiSO_4_·6H_2_O crystals obtained from the evaporative crystallization test were introduced into a 100% saturated nickel sulfate solution prepared at 50 °C. The mass ratio of the solids to the mass of the slurry, or pulp density (eqn (6)), during the repulping tests was 15%.6
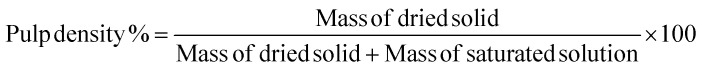


The slurry was stirred inside the jacketed reactor at 150 rpm for 70 h and the temperature inside the reactor was maintained at 50 ± 1 °C. At the end of the repulping test, nickel sulfate crystals were obtained by vacuum filtration and washed with deionized water with an amount equivalent to 10 wt% of wet-filtered crystals. The advantages of the repulping test are twofold: first, the long-time stirring of NiSO_4_·6H_2_O crystals in a pure saturated NiSO_4_ solution rinses away the mother liquor remnant on the solids that are deposited by surface adsorption/absorption; second, fluctuation of temperature (50 ± 1 °C) facilitates the recrystallization of NiSO_4_·6H_2_O, which help assess the partitioning behavior of magnesium under equilibrium conditions.

Crystallization can be described as a two-step process: (1) the transport of solutes between the mother liquor and the surface of growing crystals through a diffusion boundary layer by diffusion, followed by (2) the incorporation of the solutes into the crystals.^19^ Crystallization is typically achieved under non-equilibrium conditions owing to the slow diffusion process.^18^ This poses a problem in determining the distribution of impurity ions during crystallization. Due to the slowness of diffusion, the concentration of impurity ions in the solid and liquid phases are not at equilibrium and thus render the Berthelot-Nerst distribution law (eqn (4)) to be invalid. The possibility of achieving equilibrium between the mother liquor and the whole mass of crystals, and thus, determining the equilibrium distribution of the impurities between the solid phase and the mother solution was proved by Chlopin.^18,20^ His method was based on the recrystallization phenomenon, where the crystals were stirred in their saturated solution for a long period of time. By using this method, equilibrium between crystal and solution can be established by digesting the crystal phase sufficiently in a saturated solution to remove all concentration gradients, resulting in crystals that are formed from a solution with constant composition.^18,21^

The partitioning behavior of magnesium over liquid and solid phases at equilibrium can be described by the equilibrium distribution coefficient *D*_2/1_ (eqn (5)). At the end of the repulping test, nickel sulfate crystals were obtained by vacuum filtration and washed with deionized water with an amount equivalent to 10 wt% of wet-filtered crystals. The vacuum filter funnel was pre-heated to 50 °C to avoid undesired crystallization. After the first 70 hour repulping test, a second 24 hour repulping test was performed at 15% pulp density, and the partitioning behavior of magnesium was investigated. The second stage of repulping was performed immediately after the first repulping stage; thus, the moisture content of the wet solids from the first stage of repulping was measured using a moisture analyzer (Torbal, ATS120) to adjust the pulp density for the second repulping stage.

#### 2.3.4. Sample preparation and characterization

All liquid samples were drawn using a syringe and plastic tubing and immediately delivered to 10 mL volumetric flasks pre-filled with 5 mL of 5 wt% HNO_3_ solution using 45 μm polyethersulfone membrane syringe filters (Sarstedt AG & Co. Kg) to restrain supersaturation. The ion concentrations in the solution were measured by inductively coupled plasma optical emission spectroscopy (ICP-OES) (PerkinElmer Optima 8000). Wet solid samples were obtained from the slurry by vacuum filtration and dried using a vacuum desiccator for 48 h. The weights of the dried and wet solids were measured to determine the moisture content of the wet solids. The compositions of the solid samples were determined by first dissolving 0.5 g of solids in 50 mL deionized water and then diluting it with 5 wt% HNO_3_ solution before measuring the ion concentrations by ICP-OES. Morphological characterization was performed by scanning electron microscopy (SEM, Hitachi SU 5000). Mineralogical characterization was performed by X-ray diffraction (XRD, Rigaku Miniflex 600 Diffractometer).

#### 2.3.5. Mechanistic investigation of magnesium uptake in nickel sulfate hexahydrate crystals

A systematic approach was taken to identify the mechanism of magnesium incorporation into NiSO_4_·6H_2_O crystals. First, magnesium rejection during the evaporative crystallization test was measured to determine whether sufficient magnesium has been rejected. While there are several ways to quantify impurity rejection during crystallization, this study focuses on measuring magnesium rejection during evaporative crystallization by using the selectivity (*α*) of the process. The selectivity is described using eqn (7) and (8) as follows:7
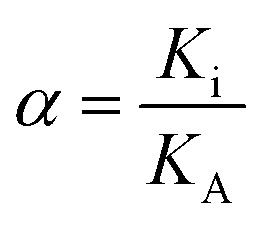
8
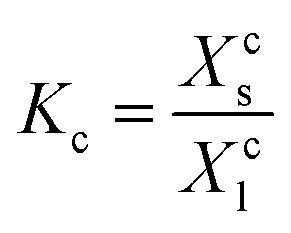
where *K*_c_ is the experimental distribution coefficient of a component, *X*^c^_s_ is the mole fraction of the component in the solid phase after crystallization, *X*^c^_l_ is the mole fraction of the component in the liquid phase after crystallization, *α* is the selectivity, *K*_i_ is the experimental distribution coefficient of the impurity ion, and *K*_A_ is the experimental distribution coefficient of the macro-component (host ion). It is important to note that *α* and 
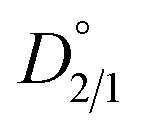
 are both dimensionless numbers that describe the partitioning behavior of the impurity ion. Here, *α* uses mole fraction as the unit of distribution coefficients, while 
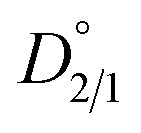
 uses concentration as the unit of distribution coefficients. However, although they may seem identical, they differ on the basis that 
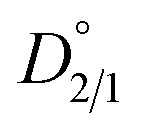
 quantifies the partitioning behavior of the impurity ion at thermodynamic equilibrium while *α* does not. *α* simply describes the partitioning behavior of impurity ions at the end of crystallization, disregarding whether the system achieved true thermodynamic equilibrium or not.

Next, the presence of agglomeration in the crystals after evaporative crystallization was determined by scanning electron microscopy (SEM). Additionally, the incorporation of magnesium by surface adsorption/absorption was investigated by surface washing to determine if a sufficient level of magnesium has been removed from the crystals.

Finally, impurity mapping was developed *via* stepwise dissolution of NiSO_4_·6H_2_O crystals to determine the extent of magnesium's lattice substitution (isomorphous substitution) into the NiSO_4_·6H_2_O crystal lattice structure. Then, 10 g of solids with a known magnesium concentration from the evaporative crystallization stage were collected for impurity mapping. The solids were suspended in 100 mL of saturated nickel sulfate solution and stirred for at least 30 minutes at room temperature (20 °C). After 30 minutes, 0.5 mL of the sample was collected using a volumetric pipette and transferred to a volumetric flask using a syringe filter. Then, 2.5 mL of deionized water was added to the suspension to dissolve about 10% wt of the solid (1 g). The suspension was stirred for further 10 minutes before taking another 0.5 mL sample, and the steps were repeated until all solids were dissolved (after a total of 10 additions of deionized water to the suspension).

Identifying the impurity incorporation mechanisms during crystallization can provide specific mitigation strategies in the process design to increase the purity of crystals.^17^ For instance, if magnesium is exclusively present on the outer surface of the crystals, the impurity rejection would be enhanced *via* constant interaction at the crystal–solution interface. Therefore, improving the filtration and washing steps after crystallization will be a good mitigation strategy to reject impurities from the crystals. On the contrary, if magnesium incorporation occurs exclusively through isomorphous substitution, washing steps alone will not be effective. Unlike surface deposition/adsorption that occurs in the later stages of crystallization, isomorphous substitution occurs during crystal growth, and consequently, the impurity is distributed uniformly throughout the bulk crystal. In such a scenario, modification must be made to the crystallization feed to decrease the concentration of magnesium in the solution during crystallization as any amount present will be likely to be incorporated into the crystal lattice.

#### 2.3.6. Selection of operating parameters

This study established operating parameters to emulate the industrial practice as closely as possible. Therefore, the initial salt concentration of the synthetic solution, the degree of evaporation (% water evaporation), pulp density (%) in repulping tests, and temperature during evaporative crystallization and repulping stages were all obtained from our industrial partner. Additionally, the operating temperature of this study (50 °C) was set to produce nickel sulfate as hexahydrate crystals. Nickel sulfate can crystallize with varying degrees of hydration, ranging from mono to heptahydrate. Based on the binary phase diagram of nickel sulfate and water, NiSO_4_·7H_2_O forms below 31.5 °C, while NiSO_4_·6H_2_O is the most stable polymorph above this temperature.^22,23^ In order to conduct evaporative crystallization at 50 °C, the pressure inside the reactor had to be adjusted by the controlled vacuum pump. Based on the saturated vapor pressure of water at different temperatures,^24^ the operational pressure for the vacuum pump was set to 123 mbar.

## 3. Results and discussion

### 3.1. Evaporative crystallization

Evaporative crystallization was carried out at 50 °C using the synthetic nickel sulfate solution with concentrations reported in Table 1. The major impurities are magnesium (0.87 g kg^−1^), copper (0.21 g kg^−1^), and zinc (0.55 g kg^−1^). As shown in Table 2, the concentration of nickel in the solution increased with the progression of evaporation. As shown in eqn (2), the nickel concentration increases the supersaturation ratio, *S*, creating a driving force for crystallization (*S* > 1). This agrees with the working principles of evaporative crystallization. It is also worth noting that once the vacuum was removed after 60% water evaporation, the concentration of nickel and all other solutes in the solution remained constant, indicating that the change in the concentration of the solutes was due to evaporation.

**Table 2:** Composition of the mother liquor during evaporative crystallization of nickel sulfate

Sample	[g kg^−1^ solution]						
	Ca	K	Na	Cu	Mg	Ni	Zn
25% evaporation	0.02	0.02	0.01	0.16	1.00	99.35	0.78
50% evaporation	0.04	0.03	0.01	0.20	1.32	134.06	1.03
60% evaporation	0.03	0.04	0.02	0.31	1.76	137.84	1.33
After stirring overnight	0.03	0.04	0.02	0.32	1.72	134.13	1.33
Mother liquor	0.04	0.04	0.02	0.31	1.73	137.73	1.32
Wash solution	0.03	0.01	0.00	0.06	0.69	104.55	0.54

Table 3 presents the composition of the solids at 60% water evaporation, after overnight stirring, and after vacuum filtration (pre-wash and post-wash with deionized water). The effect of overnight stirring of slurry on the impurity uptake of nickel sulfate crystals was investigated. As shown in Table 3, overnight stirring did not affect the impurity concentrations of the solids; the magnesium concentration remained constant before and after stirring overnight (1.52 *vs.* 1.55 g kg^−1^). Table 3 also shows that washing the solids with deionized water (10 wt% of wet solids after vacuum filtration) after vacuum filtration did not change the concentration of the magnesium significantly (1.30 *vs.* 1.26 g kg^−1^).

**Table 3:** Composition of the solids after evaporative crystallization of nickel sulfate

Sample	[g kg^−1^ solid]						
	Ca	K	Na	Cu	Mg	Ni	Zn
After 60% evaporation	0.05	0.01	0.00	0.16	1.52	235.07	1.26
After stirring overnight	0.08	0.02	0.00	0.18	1.55	229.73	1.29
Pre-wash solids (after vacuum filtration)	0.07	0.08	0.00	0.11	1.30	228.49	1.08
Post-wash solids (after vacuum filtration)	0.07	0.09	0.00	0.11	1.26	229.91	1.06

The extent of magnesium rejection during evaporative crystallization was measured using the selectivity coefficient (*α*, eqn (7)).^17^Table 4 presents the experimental distribution coefficient of magnesium (impurity ion) and nickel (host ion), and the resulting selectivity coefficient. Ideally, the selectivity coefficient, *α*, should be as low as possible; the *α* value of 0 signifies complete rejection of the impurity ion in the solid phase, while the *α* value of 1 signifies that the impurity ion and the host ion have an equal preference to be taken up by the crystalline material. If there is no purity specification set, it is recommended to aim for *α* = 0.05.^17^ The experimental *α* value in this work was calculated to be 0.45, which is above the desired threshold. This indicates inadequate rejection of magnesium during the crystallization of NiSO_4_·6H_2_O, which is in accordance with the previous findings reported in the literature.^9,12,22^

**Table 4:** Experimental distribution coefficient and selectivity of evaporative crystallization

Host ion (A)	Impurity (i)	*K*_i_	*K*_A_	*α* = *K*_i_/*K*_A_
Ni^2+^	Mg^2+^	0.46	1.02	0.45

**Fig. 2:**
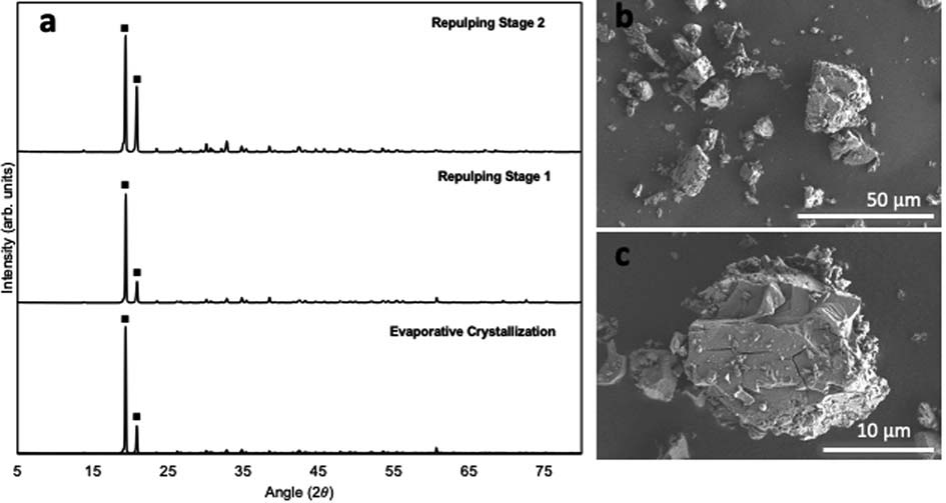
(a) XRD diffractogram of nickel sulfate crystals obtained after evaporative crystallization, repulping stage 1, and repulping stage 2. (b) and (c) SEM images of crystals obtained after evaporative crystallization. Scale bar: (b) 50 μm and (c) 10 μm.

The XRD diffractogram of the solid after evaporative crystallization and the subsequent two repulping tests are presented in Fig. 2a. As shown, the two characteristic peaks, or the two highest intensity peaks, appear at 19.4° and 20.9° in the solid samples. These two values of the characteristic peaks (19.4° and 20.9°) match the finding of a previous study, which identified that these two peaks are characteristic of *α*-NiSO_4_·6H_2_O.^22^ The morphology of the nickel sulfate crystals produced in this study also corresponds well with the previous study that reported the shape of *α*-NiSO_4_·6H_2_O to be thick plates or short-prismatic crystals (Fig. 2b and c).^25^ Based on these results, it can be concluded that the phase of nickel sulfate crystals obtained in this study is indeed the hexahydrate polymorph, which is the most commonly used hydrated nickel sulfate salt in the battery-making industry.

### 3.2. Effect of repulping on the purity of nickel sulfate hexahydrate crystals

The effect of repulping on the purity of NiSO_4_·6H_2_O was investigated at a pulp density of 15%. As previously discussed, repulping can help increase the purity of the crystals by removing the impurities that are deposited on the surface and facilitating the recrystallization of crystals *via* long-time stirring of crystals in their saturated solution. During the first stage of repulping, samples were taken at 5, 45, 60, 180, and 4200 min (70 h) to determine the distribution coefficient and to investigate whether equilibrium has been reached between the crystals and the solution liquid phase.

As shown in Table 5, there is a general decrease in the concentration of impurities in the solids after the 70 h repulping test. The concentration of copper, magnesium, and zinc decreased by 18%, 72%, and 72%, respectively in the unwashed solids. Washing the crystals with deionized water further decreased the concentration of impurities. Copper, magnesium, and zinc concentrations decreased by 11%, 37%, and 46%, respectively. The stagnant decrease in the concentration of magnesium (0.35 g kg^−1^*vs.* 0.22 g kg^−1^) after washing with deionized water indicates that the residual magnesium in the crystals after repulping can be due to the incorporation of magnesium inside the crystal lattice, which cannot be removed through surface washing.^17^

**Table 5:** Composition of solid and liquid phases in the repulping stage (1st) obtained from ICP-OES measurements

Element	Crystallization stage	Repulping stage 1				
	Dry crystal (g kg^−1^)	100% saturated nickel sulfate solution (g kg^−1^)	Liquid after 70 h test (g kg^−1^)	Dry repulped crystal pre-wash (g kg^−1^)		Dry repulped crystal post-wash (g kg^−1^)
K	0.09	0.00	0.00		0.12	0.03
Ca	0.07	0.01	0.01		0.06	0.06
Cu	0.11	0.00	0.02		0.09	0.08
Mg	1.26	0.00	0.17		0.35	0.22
Na	0.00	0.01	0.01		0.01	0.00
Ni	229.91	144.65	148.26		232.74	233.23
Zn	1.06	0.00	0.13		0.30	0.19

**Fig. 3:**
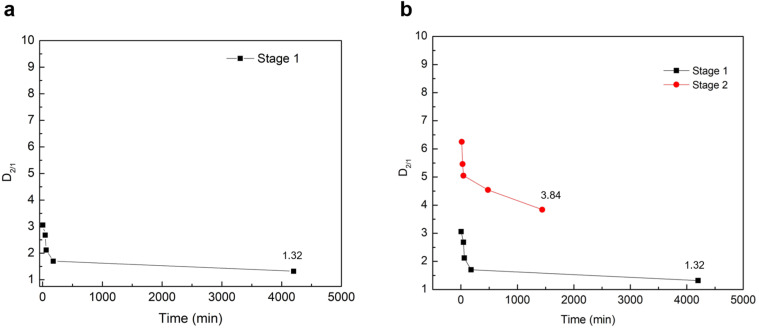
Effect of time on the distribution coefficient during (a) the first-stage repulping and (b) the second-stage repulping experiments.

To quantify the partitioning behavior of magnesium during the 70 h repulping test, the values of the distribution coefficients are plotted as a function of time using eqn (5). Fig. 3a shows the experimental value of 
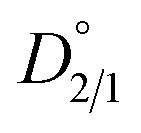
 from the first stage of repulping to be 1.32. The value of 
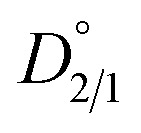
 close to 1 indicates that magnesium and nickel have a roughly equal preference to be taken up by the crystal lattice structure at equilibrium. In other words, Mg^2+^ and Ni^2+^ partition into the solid and liquid phases with approximately equal ratios at equilibrium. From Fig. 3a, it is also evident that the value of *D*_2/1_ decreases with the increase in time before achieving equilibrium. This matches the experimental results on determining the equilibrium distribution coefficient by using the method of long-time stirring of contaminated crystals in a pure saturated solution.^26,27^

This trend of a gradual decrease in the concentration of Mg^2+^ can be explained by the concentration gradient that exists for Mg^2+^ at the diffusion boundary layer between the mother liquor and the surface of the growing crystals. As previously noted, the transport of solutes between the mother liquor and the surface of growing crystals occurs through a diffusion boundary layer by diffusion during crystallization.^19^ In other words, the diffusive boundary layer at the liquid–solid interface serves as a mode of mass transfer *via* molecular diffusion between the growing crystals and the mother liquor. When repulping the crystals with a saturated, pure NiSO_4_ solution, the concentration of Mg^2+^ at the solid interface is higher than at the liquid side. Therefore, there is a net mass transfer of Mg^2+^*via* diffusion from the solid interface into the mother liquor through the diffusive boundary layer until it reaches equilibrium, where it no longer experiences a net transfer of mass between the two phases. Correspondingly, Table 6 shows that the distribution coefficient of Ni^2+^, K_1_, remains relatively constant compared with the distribution coefficient of Mg^2+^, K_2_, where it decreases until it reaches equilibrium. Thus, it can be concluded that the decrease in the values of *D*_2/1_ in Table 6 can be primarily due to the decrease in the concentration of magnesium in the solid phase.

**Table 6:** *K*_1_, *K*_2,_ and *D*_2/1_ values for the first repulping stage at different time intervals

Time (min)	*K*_1_	*K*_2_	*D*_2/1_ = *K*_2_/*K*_1_
5	1.68	5.15	3.06
45	1.70	4.56	2.68
60	1.69	3.58	2.12
180	1.62	2.76	1.70
4200	1.57	2.07	1.32

### 3.3. Effect of multi-stage repulping

The effect of multi-stage repulping on the impurity removal of NiSO_4_·6H_2_O crystals was investigated. As shown in Table 7, while the removal of magnesium did occur in the second stage, the extent of removal was significantly less than that in the first stage of repulping. For instance, there was only a small decrease in the concentration of magnesium (0.22 g kg^−1^*vs.* 0.20 g kg^−1^) at the end of the repulping test, before washing with deionized water (9% decrease in the concentration). After washing with deionized water, the magnesium concentration also did not change significantly (0.20 g kg^−1^ to 0.19 g kg^−1^), which can be explained by the isomorphous substitution of magnesium inside the crystal lattice. Compared with the first-stage repulping test, the removal of magnesium was much slower in the second stage of the repulping test, thus taking a longer time to reach equilibrium in the second stage. This trend agrees with the resulting value of *D*_2/1_ at the end of the second repulping stage, 3.84, which is significantly higher than the equilibrium distribution coefficient determined in the first stage of the repulping test (Fig. 3b). The discrepancy in the distribution coefficient values between the two repulping stages indicates that the second stage of repulping did not achieve thermodynamic equilibrium within the given 24 h test; under equilibrium conditions, the respective concentrations of magnesium may be different, but the distribution coefficient should be the same since it is a ratio of Mg^2+^ concentrations in the solid and liquid phases at equilibrium. This observed discrepancy can be explained by Fick's first law of diffusion, which states that the diffusion flux and diffusion rate are directly proportional to the concentration gradient.^28^ Since pure, saturated NiSO_4_ liquid solution ([Mg^2+^] = 0 mg L^−1^) was used in both stages of repulping, the concentration gradient across the diffusion boundary layer is higher in the first stage of repulping than in the second stage. This is because the crystals introduced in the first repulping stage were more contaminated (*i.e.*, a higher concentration of Mg^2+^) than the subsequently purified crystals employed in the second repulping stage. Therefore, this difference in the concentration gradient of Mg^2+^ resulted in a higher diffusive flux of Mg^2+^ in the first stage of repulping. This is evident in Table 8, which shows that the K_2_ values from the second stage of repulping decrease much slower than the K_2_ values from the first stage of repulping, as shown in Table 6. This explains why the first stage of repulping reached equilibrium in this study, while the second stage of repulping did not.

**Table 7:** Composition of solid and liquid in the repulping stage (2nd) measured by ICP-OES

Element	Repulping stage 1	Repulping stage 2				
	Dry crystal (g kg^−1^)	100% saturated nickel sulfate solution (g kg^−1^)	Liquid after 24 h test (g kg^−1^)	Dry repulped crystal pre-wash (g kg^−1^)		Dry repulped crystal post-wash (g kg^−1^)
K	0.03	0.00	0.00		0.00	0.01
Ca	0.06	0.01	0.01		0.07	0.08
Cu	0.08	0.00	0.00		0.04	0.04
Mg	0.22	0.00	0.03		0.20	0.19
Na	0.00	0.01	0.01		0.00	0.00
Ni	234.45	144.65	139.23		232.15	236.60
Zn	0.19	0.00	0.02		0.17	0.15

**Table 8:** *K*_1_, *K*_2_, and *D*_2/1_ values for the second repulping stage at different time intervals

Time (min)	*K*_1_	*K*_2_	*D*_2/1_ = *K*_2_/*K*_1_
15	1.66	10.36	6.25
30	1.65	9.01	5.46
45	1.66	8.39	5.05
480	1.65	7.47	4.54
1440	1.67	6.40	3.84

**Fig. 4:**
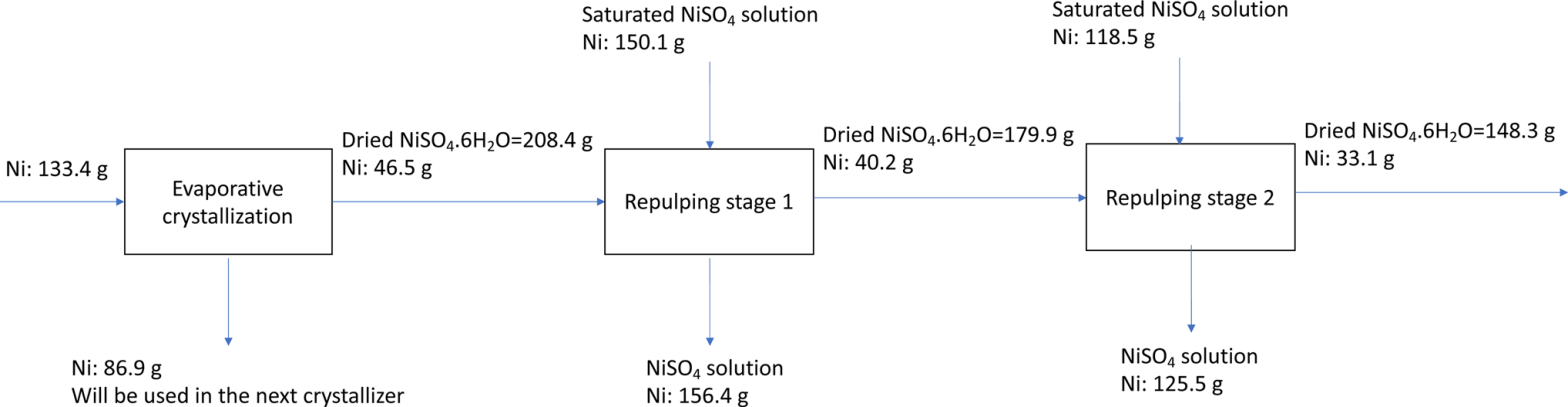
Block flow diagram of the process and Ni mass balance.

To determine the Ni retaining ratio using two-stage repulping, the Ni mass balance was calculated, and the results are presented in the block flow diagram in Fig. 4. As shown, the Ni retaining ratio in repulping stage 1 is 86% and in repulping stage 2 is 82%. If we consider two repulping stages, the total Ni retaining ratio is 71%.

### 3.4. Mechanistic investigation of magnesium incorporation

The selectivity value of evaporative crystallization (*α* = 0.45) indicates that sufficient rejection of magnesium was not achieved, which necessitates an investigation of possible incorporation mechanisms of magnesium into NiSO_4_·6H_2_O crystals.

**Fig. 5:**
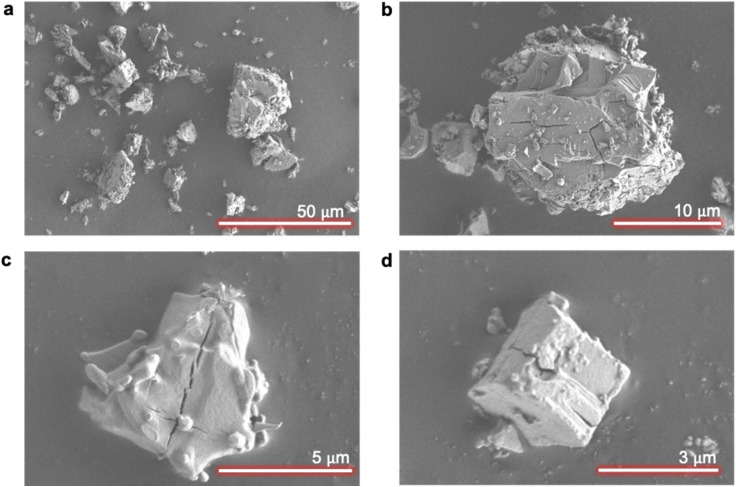
SEM images of crystals from evaporative crystallization. Scale bars: (a) 50 μm, (b) 10 μm, (c) 5 μm, and (d) 3 μm.

Agglomeration was the first impurity uptake mechanism that was investigated. Impurity uptake through agglomeration occurs when small particles aggregate during crystallization and trap the impurity-laden mother liquor between the growing particles.^29^ As shown in Fig. 5, it is evident that evaporative crystallization employed in this study resulted in well-separated solids with no signs of aggregations or agglomeration; thus, it can be concluded that agglomeration is not one of the impurity uptake mechanisms of magnesium into NiSO_4_·6H_2_O crystals.

Next, this study investigated whether surface adsorption/deposition was a primary mechanism of magnesium incorporation. Impurity uptake *via* surface deposition/adsorption can occur when the residual impurity-laden mother liquor is not completely washed off from the surface of the crystals, thereby affecting the purity of the resulting crystals. Additionally, the adsorption of impurities can occur when the impurities have high affinities for the surface of the crystals. The extent of surface adsorption/deposition of magnesium can be investigated by washing the solids with a saturated NiSO_4_ solution. If there is a significant decrease in the concentration of the impurity (>50%) after washing, it is concluded that the primary mechanism of impurity incorporation is surface deposition and adsorption.^17^ As shown in Table 5, it has already been determined in this study that the magnesium concentration decreased by 72% during the first repulping test. While this suggests that surface deposition may be a major mechanism in which magnesium is incorporated into NiSO_4_·6H_2_O crystals, further investigation was needed to determine whether surface adsorption is the only mechanism of magnesium uptake or if there are other mechanisms involved. Additionally, the repulping stage employed in this study had two combined effects on the purity of resulting crystals, *i.e.*, surface washing and recrystallization. Therefore, it cannot be determined how much of the observed 72% concentration decrease can be attributed to surface washing alone.

**Fig. 6:**
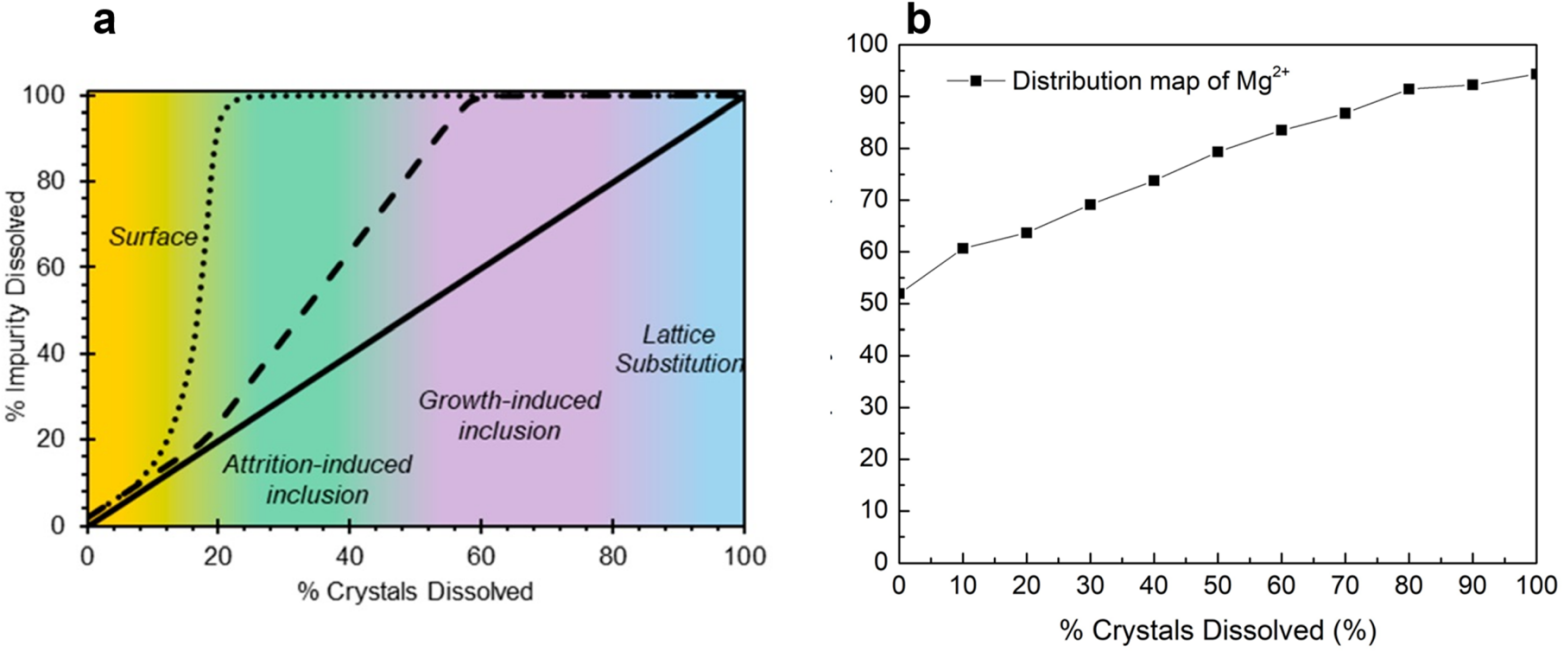
Impurity distribution map from (a) literature^17^ and (b) experimental work in this study.

Finally, this study investigated inclusion (attrition-based and growth-based) and lattice substitution as the possible impurity uptake mechanisms for Mg^2+^. This was done by adopting the impurity distribution map technique reported in the literature.^17^ The impurity distribution map can be plotted *via* stepwise dissolution of crystals in a saturated NiSO_4_ solution and measuring the percent increase in the Mg^2+^ concentration in the saturated NiSO_4_ solution. The advantage of using an impurity distribution map is its ability to visually represent various impurity uptake mechanisms. For instance, if the impurities are exclusively present on the outer layers of the crystals (*via* surface deposition and/or adsorption), there will be a sharp increase in the concentration during the initial stirring steps, followed by the almost complete release of impurities into the solution at around 20% dissolution, and plateaus for the remainder of solvent addition steps (Fig. 6a, surface). In contrast, there would be a steadier increase in the impurity concentration for attrition-induced and growth-induced inclusions (Fig. 6a, inclusions). If there is a linear relationship between the percent crystals dissolved and the percent impurity dissolved, it can be concluded that the impurities are distributed uniformly throughout the bulk crystal, and thus, incorporation has likely occurred *via* lattice substitution (Fig. 6a, lattice substitution). Growth-induced inclusion can occur because of the inclusion of impurity-laden mother liquor during rapid crystal growth while attrition-induced inclusion can occur from high-energy agitation that results in the inclusion of impurity-laden mother liquor into the colliding particles.^17^ Lattice substitution, as previously noted in this study, occurs through isomorphous substitution.

**Fig. 7:**
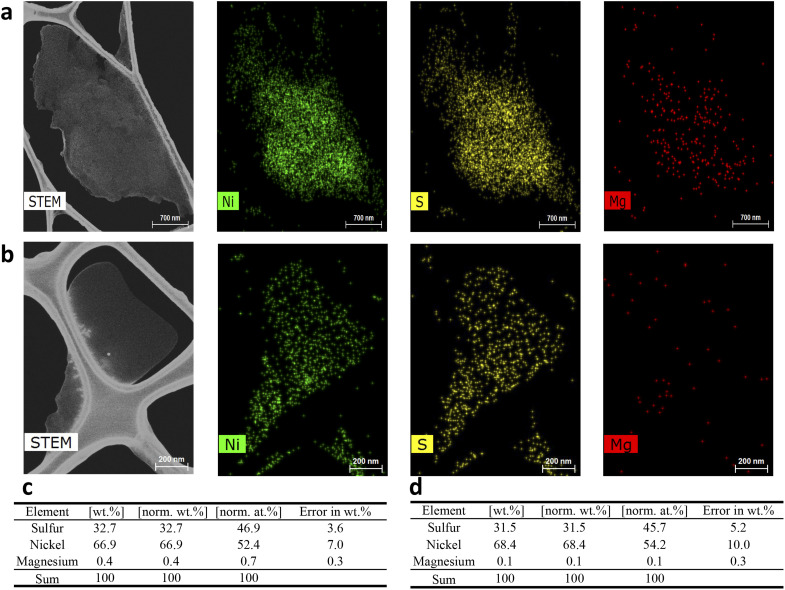
STEM-EDX analysis of crystals after (a) evaporative crystallization and (b) two-stage repulping tests. Elemental analysis of sulfur, nickel, and magnesium using SEM-EDX for (c) crystals after evaporative crystallization and (d) after two-stage repulping tests. Scale bar: (a) 700 nm and (b) 200 nm.

The total mass of Mg^2+^ in the solids employed for the impurity mapping was determined to be 16.79 mg. As shown in Fig. 6b, initial stirring of the suspended nickel sulfate crystals in the saturated NiSO_4_ solution—before the solvent addition steps—resulted in about 50% of the total mass of Mg^2+^ in the solids being released into the mother liquor (8.72 mg). This strongly suggests that a significant amount of Mg^2+^ is located on the surface of the crystals and is incorporated *via* the surface deposition/adsorption mechanism. This provides additional support for the results from the repulping stage, where a notable amount of magnesium was removed by surface washing. The surface contamination of magnesium can be further supported by STEM-EDX analysis (Fig. 7). The elemental mapping of nickel sulfate hexahydrate crystals after evaporative crystallization (Fig. 7a) and after two-stage repulping (Fig. 7b) shows that the magnesium content in the crystal after evaporative crystallization is higher than that in the crystal after two stages of repulping. The elemental compositions for both cases are shown in Fig. 7c and d.

Additionally, Fig. 6b shows that the impurity concentration in the solution increases linearly with the increase in the percent dissolution of crystals, which confirms that magnesium is incorporated into the crystal by lattice substitution, as suggested by the linear relationship of the impurity distribution map (Fig. 6a, lattice substitution). Based on these results, it can be concluded that there are two mechanisms for magnesium uptake during NiSO_4_·6H_2_O, *i.e.*, surface deposition/adsorption and lattice substitution.

## 4. Conclusions

A fundamental investigation on the purification of NiSO4·6H2O crystals by coupling evaporative crystallization and repulping stages has been performed. Additionally, a systematic approach was followed to determine the magnesium incorporation mechanism into NiSO_4_·6H_2_O crystals. Experimental results indicate that evaporative crystallization does not sufficiently reject magnesium during NiSO_4_·6H_2_O crystallization. Additionally, overnight stirring after evaporative crystallization does not affect the magnesium concentration in the NiSO_4_·6H_2_O crystals. It was experimentally determined that repulping with 100% saturated nickel sulfate solutions following evaporative crystallization results in magnesium removal. In the first stage, there was a 72% decrease in the concentration of magnesium, while the second stage was less effective, and only a 9% decrease in the concentration of magnesium was achieved. The difference in the purification efficiencies of these two repulping stages can be explained by the experimentally determined distribution coefficients.

The equilibrium distribution coefficient 
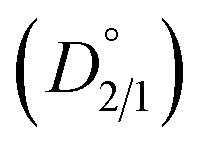
 of magnesium during the first stage of repulping was determined to be 1.32. With a value close to 1, this suggests that magnesium (impurity) and nickel (host atom) have an equal preference to be taken up by the crystal lattice at 50 °C (equilibrium condition). Upon additional repulping at 50 °C, the distribution coefficient (*D*_2/1_) was 3.84, which indicates that it did not achieve equilibrium owing to the smaller diffusion flux of the magnesium ion across the diffusion boundary layer between the solid interphase and bulk liquid compared with the first repulping stage, resulting in a lower magnesium removal rate. Repulping is effective in removing impurities because the magnesium incorporation mechanism comprises both isomorphous substitution and surface deposition/adsorption, which is confirmed using the systematic approach taken for mechanistic investigation.

## Author contributions

Kyoung Hun Choi: formal analysis, investigation, methodology, writing – original draft, writing – review & editing. Gisele Azimi: conceptualization, formal analysis, funding acquisition, investigation, methodology, project administration, resources, supervision, validation, writing – review & editing.

## Conflicts of interest

The authors declare that they have no known competing financial interests or personal relationships that could have appeared to influence the work reported in this paper.

## Supplementary Material
